# Device Physics and Design Principles of Mixed‐Dimensional Heterojunction Perovskite Solar Cells

**DOI:** 10.1002/smsc.202300188

**Published:** 2024-01-20

**Authors:** Yuqi Zhang, Zhenhai Yang, Tianshu Ma, Zhenhai Ai, Yining Bao, Luolei Shi, Linling Qin, Guoyang Cao, Changlei Wang, Xiaofeng Li

**Affiliations:** ^1^ School of Optoelectronic Science and Engineering & Collaborative Innovation Center of Suzhou Nano Science and Technology Soochow University Suzhou 215006 China; ^2^ Key Lab of Advanced Optical Manufacturing Technologies of Jiangsu Province & Key Lab of Modern Optical Technologies of Education Ministry of China Soochow University Suzhou 215006 China

**Keywords:** 2D/3D perovskite, carrier transport, perovskite solar cells, photoelectric simulations

## Abstract

Mixed‐dimensional perovskites possess unique photoelectric properties and are widely used in perovskite solar cells (PSCs) to improve their efficiency and stability. However, there is a pressing need for a deeper understanding of the physical mechanisms and design principles of mixed‐dimensional PSCs, as such knowledge gaps impose restrictions on unlocking the full potential of this kind of PSC. Herein, a 2D/3D PSC is employed as an example to clarify the working mechanism of mixed‐dimensional PSCs from the perspective of device physics and elaborate on the design rules of high‐efficiency mixed‐dimensional PSCs. Detailed simulation results indicate that the insertion of a layer of 2D perovskite between the 3D perovskite and the hole transport layer (HTL) can significantly reduce the recombination at the HTL/perovskite interface, and PSCs with a 2D/3D perovskite structure exhibit higher tolerance to material selectivity compared with their 3D counterparts. Additionally, the 2D/3D perovskite design can slow down ion migration and accumulation processes, thereby easing the hysteresis behavior of 2D/3D PSCs. Moreover, it is also found that the 2D/3D perovskite structure has a more pronounced effect on improving the efficiency of wide‐bandgap PSCs. Overall, this work sheds new light on mixed‐dimensional PSCs, enabling better guidance for designing high‐efficiency PSCs.

## Graphical abstract

By conducting a rigorous photoelectric simulation, the intrinsic mechanisms behind the efficiency improvement of 2D/3D perovskite solar cells (PSCs) are clarified and the dependence of device performance on material selectivity is studied. The 2D/3D perovskite design can slow down ion migration and accumulation processes, and the 2D/3D perovskite structure is more significant for improving the performance of the wide‐bandgap PSCs.

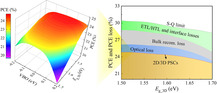

## 1 Introduction

Perovskite solar cells (PSCs) have gained considerable attention due to their notable advantages in power conversion efficiency (PCE) and fabrication cost, making them a promising candidate for commercialization. Continued progress in material selection, film quality, interface modification, and device integration has recently enabled PSCs to achieve a new record efficiency of 26.1%.^[^ ^1^ ^]^ Sustaining efficiency improvements of PSCs to catch up with mainstream crystalline silicon (c‐Si) SCs (with a record efficiency of 26.81%) remains critical to enhance their competitive edges in the photovoltaic (PV) community.^[^ ^2^ ^]^ However, further increasing the efficiency of PSCs remains challenging, as it requires highly complex fabrication processes and stringent material selection criteria.

In normal *n‐i‐p* or *p‐i‐n* structured PSCs, electron‐/hole‐transport layers (ETLs/HTLs) play a crucial role in passivating interface defects and extracting charge‐carriers within the perovskite layers, although additional interface passivation/functional layers have been introduced to alleviate the pressure or enhance the effect of ETLs/HTLs.^[^ ^3^, ^4^ ^]^ On one hand, the use of ETLs/HTLs alone (i.e., without additional passivation/functional layers) poses a challenge in ensuring simultaneous good passivation and high‐efficiency extraction of charge‐carriers due to their conflicting effects, holding back a plethora of opportunities for screening functional/passivation materials.^[^ ^5^ ^]^ On the other hand, the presence of additional passivation/functional layers will also bring many problems, such as increasing contact/bulk resistances or inducing unfavorable energy‐band bending, which, as has been widely confirmed, will degrade the efficiency and aggravate the stability of the related devices.^[^ ^6^ ^]^ Consequently, a technical innovation in device structures/designs that target the perovskite layer itself, rather than simply introducing additional functional/passivation layers, is expected to offer a new driving force for PSCs to unlock their full potentials with higher efficiencies and provide an expansive window for processing conditions and material selectivity.^[^ ^7^, ^8^ ^]^

High‐efficiency c‐Si SCs with various advanced design concepts can serve as a reference. Taking a typical c‐Si technology as an example, tunnel oxide passivating contact (TOPCon) SCs rely on ultra‐thin SiO_x_ (silicon oxide) films associated with heavily‐doped polysilicon layers, flexibly mediated by in‐diffusion doping, to balance passivation and contact properties, and thus afford high device efficiencies. The in‐diffusion impurity underneath the c‐Si surface serves as polar dopants to form a high/low junction between c‐Si substrates, offering an assisted electric field to suppress minority carrier recombination and accelerate majority carrier extraction.^[^ ^9^ ^]^ Inspired by the ingenious designs of c‐Si devices, researchers have attempted various strategies for PSCs, including homojunction, heterojunction, bandgap‐gradient, mixed‐dimensional perovskite, etc.^[^ ^10^, ^11^, ^12^, ^13^ ^]^ Among these advanced designs, mixed‐dimensional perovskite, such as 2D/3D perovskite, has become a research hotspot due to its unique advantages of high compatibility in the fabrication process and high‐efficiency potential. The mixed‐dimensional perovskite structure offers many benefits. First, the low‐dimensional perovskite (e.g., 2D perovskite) formed by incorporating specific organic spacer cations usually has a higher bandgap, providing an opportunity to regulate the energy level alignment and electric field intensity to improve passivation quality. Second, the low‐/high‐dimensional perovskite interface often has fewer defects due to the compatibility of preparation methods and the absence of sputtering damage.^[^ ^13^ ^]^ More importantly, mixed‐dimensional PSCs are usually more stable due to the fact that the low‐dimensional perovskite phases can protect the high‐dimensional perovskite from being damaged by subsequent film deposition or ambient factors.^[^ ^14^ ^]^ Benefiting from these advantages, 2D/3D PSCs often show higher efficiency and better stability than their counterparts without 2D perovskite, in which the representative works are summarized in Table S1 and Figure S1, Supporting Information. Despite many significant achievements, the full potential of mixed‐dimensional PSCs has yet to be realized, partially due to the unclear working mechanisms and design principles required to screen and build efficient mixed‐dimensional perovskite structures for PSCs.

In this study, we aim to clarify the intrinsic mechanisms and design rules of mixed‐dimensional PSCs by conducting rigorous photoelectric simulations. Using a typical *n‐i‐p* PSC as an example, we investigate the photoelectrical performance of 2D/3D PSCs in detail and make the following key observations: 1) inserting a layer of 2D perovskite between the 3D perovskite and the HTL could significantly reduce the recombination at the HTL/perovskite interface, which is the origin of efficiency improvement of 2D/3D PSCs; 2) compared with the normal 3D PSCs, the 2D/3D PSCs exhibit higher tolerance to material selectivity. This is noteworthy because, even when incorporating transport layers with smaller bandgaps or unfavorable energy alignments, 2D/3D PSCs can achieve higher PCE values, suggesting the feasibility of integrating a broader range of transport layers to construct high‐efficiency 2D/3D PSCs; 3) the 2D/3D perovskite design with a relatively low electric field within 3D perovskite layer could slow down ion migration and accumulation processes, thereby suppressing the hysteresis behavior of 2D/3D PSCs; and 4) the 2D/3D perovskite structure is more effective in improving the performance of wide‐bandgap PSCs. This study provides a deeper insight into the working mechanisms and design principles of 2D/3D PSCs, which is beneficial for designing high‐efficiency PSCs.

## 2 Results and Discussion

**Figure 1:**
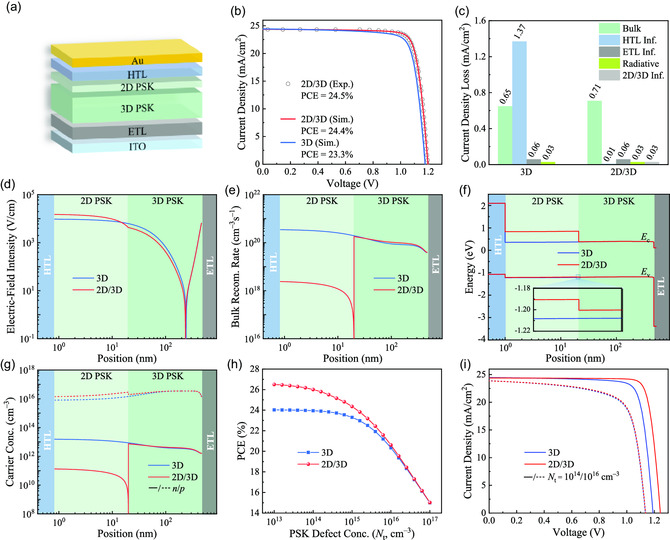
a) Schematic diagram of 2D/3D PSC used for this study and b) the corresponding *J–V* curves from simulation and experiment, where the experimental results were extracted from reference.^[^ ^15^ ^]^ c) Distributions of the simulated current density losses and the position‐dependent distributions of d) electric field intensity, e) bulk recombination rate, f) energy level, and g) carrier concentration of 3D and 2D/3D PSCs under MPP‐like condition. h) The dependence of bulk defect concentration of perovskite layer on device PCE and i) the representative *J–V* curves of 3D and 2D/3D PSCs under *N*_t_ = 10^14^ and 10^16^ cm^−3^.

In this study, we focus on a typical *n‐i‐p* PSC, where a layer of 2D perovskite is inserted between the 3D perovskite and the HTL interface, as illustrated in **Figure** **1**a. Based on the experimental results conducted by Sidhik et al., we carry out comprehensive simulations, and fit our simulated current–voltage (*J–V*) curve with the experimental one to confirm the reliability of our simulation model.^[^ ^15^ ^]^ The simulation details are provided in the Experimental Section, and the parameters used for this simulation are listed in Table S2, Supporting Information. To reproduce the experimental *J–V* curve, we conduct photoelectric simulations using the identical parameters as those in the experiment, including perovskite bandgap/thickness, ETL, HTL, electrode materials, etc. Figure 1b depicts the experimental and simulated *J–V* curves, indicating that the simulated plot (red line) fits well with the experiment one (black circles). This provides a basis for our simulation and subsequent theoretical analysis. In this study, we also consider the counterpart with only 3D perovskite, and the corresponding *J–V* curve of 3D PSC (blue line) is shown in Figure 1b. From these plots, we can see that the 2D/3D PSC with a PCE of 24.4% exhibits a higher device efficiency than that of the 3D PSC (23.3%). Specifically, the PV parameters are listed in Table S3, Supporting Information, indicating that the improved fill factor (FF) and open circuit voltage (*V*_oc_) are the main factors contributing to the superior performance of the 2D/3D PSC.

To uncover the underlying reasons for the performance improvement of 2D/3D PSC, we investigate the distributions of current density losses of two related cases under the maximum power point (MPP)‐like condition. The simulated results in Figure 1c and S2, Supporting Information indicate that the current density loss at the HTL/perovskite interface for 2D/3D PSCs is much lower than that in 3D PSCs, indicating that the presence of the 2D perovskite can significantly reduce recombination loss at the HTL/perovskite interface. We further clarify the underlying mechanism of the 2D/3D design by analyzing the position‐dependent electric field intensity, energy level, carrier concentration, and bulk recombination profiles. As demonstrated in Figure 1d, the 2D/3D PSC exhibits a higher electric field at the 2D/HTL interface and 2D bulk region compared to the 3D counterpart, which is one of the main reasons for the lower recombination loss at the 2D/HTL interface, as shown in Figure 1c. Unfortunately, the 2D/3D PSC exhibits a lower electric field intensity at the 3D perovskite layer in comparison to the 3D PSC, thus resulting in slightly increased recombination at the 3D perovskite layer, as shown in Figure 1c,e. Despite this disadvantage (i.e., the reduced electric field at 3D bulk for 2D/3D PSC), the performance of the 2D/3D PSC can be compensated by the 2D/3D heterojunction structure. Specifically, the 2D/3D structure creates an energy cliff at the 2D/3D interface, as shown in Figure 1f, which can promote hole (majority carrier) transport and suppress electron (minority carrier) recombination by controlling carrier distributions, as displayed in Figure 1g. Overall, we can conclude that the 2D/3D structure of this study can provide a strong electric field at the 2D/HTL interface to regulate carrier concentration, and ultimately be beneficial for suppressing carrier recombination. In addition, the dependence of perovskite defect concentration on device performance is also studied, with the corresponding results demonstrated in Figure 1h. It is evident that the 2D/3D PSCs show higher PCE values than 3D PSCs until the perovskite defect concentration (*N*_t_) is higher than 10^16^ cm^−3^, which means that 2D/3D PSCs have higher tolerance to *N*_t_ if it is less than 10^16^ cm^−3^. In most cases, perovskite has a measured *N*_t_ of 10^14^–10^15^ cm^−3^, implying that the 2D/3D structure could improve device performance within the experimental range. Moreover, the corresponding *J*–*V* curves under two typical *N*_t_ cases (i.e., 10^14^ and 10^16^ cm^−3^) are plotted in Figure 1i. It is evident that when *N*_t_ = 10^16^ cm^−3^, the 2D/3D PSC exhibits nearly identical *J*–*V* behavior in contrast to the 3D PSC, but when *N*_t_ = 10^14^ cm^−3^, the 2D/3D PSC shows a much higher *V*_oc_, consistent with the experiment observations.^[^ ^16^, ^17^ ^]^

**Figure 2:**
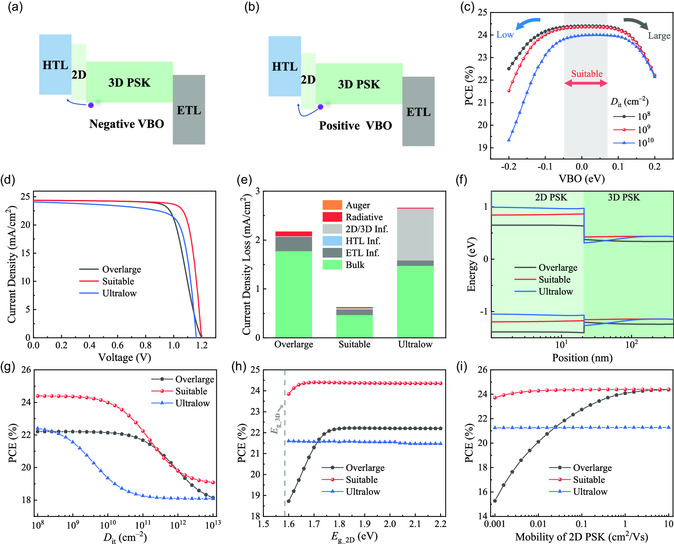
Schematic diagrams of 2D/3D PSCs with a) the negative VBO and b) the positive VBO of 2D/3D perovskite interface. c) Dependence of the VBO values on device PCE under the different *D*_it_ cases, i.e., *D*_it_ = 10^8^, 10^9^, and 10^10^ cm^−2^. d) The representative *J–V* curves, e) the corresponding current density loss distributions, and f) the energy band structures at MPP‐like condition under the different VBO cases, where *D*_it_ is fixed at 10^8^ cm^−2^. PCE of 2D/3D PSCs as a function of g) the defect density of 2D/3D perovskite interface, h) 2D perovskite bandgap, and i) 2D perovskite mobility under the different VBO cases. Here, the overlarge, suitable, and ultralow represent the cases of VBO = 0.2, 0.02, and –0.2 eV, respectively.

In the previous section, we confirmed that the 2D/3D structure can significantly improve the performance of PSCs in specially designed cases. It is of great significance to unlock the full potential of such a 2D/3D PSC toward higher device efficiency and clarify the relevant mechanisms for performance improvement. In this section, we investigate the effect of the valence band offsets (VBO) of 2D and 3D perovskite on device performance, and the schematic diagrams of the negative and positive VBO cases are illustrated in **Figure** **2**a,b, respectively. The variation of device PCE with VBO under three typical defect densities (*D*_it_) of 2D/3D perovskite interface is plotted in Figure 2c. We can conclude from this figure that the device PCE can be well‐maintained over a large range of VBO, i.e., –0.05 eV < VBO < 0.1 eV, meaning that the 2D/3D PSC has a high tolerance for the VBO of 2D/3D perovskite. However, an obvious decrease in PCE can be observed if the VBO is too high or too low. The representative *J*–*V* curves are displayed in Figure 2d, which reveal that devices with an overlarge energy structure (i.e., VBO = 0.2 eV) show a significant decrease only in FF, while the ultralow case (i.e., VBO = –0.2 eV) has a notable reduction in both FF and *V*_oc_. To account for these observations, the distributions of current density losses and energy band structures are checked, as shown in Figure 2e,f, respectively. For the overlarge VBO case, a high energy barrier for hole transport at the 2D/3D perovskite interface is formed as demonstrated in Figure 2f, which thus leads to the accumulation of holes, especially in the region near the 2D/3D perovskite interface (Figure S4, Supporting Information), an increase in bulk recombination of the perovskite layer, and a decrease in the *FF* of devices. It is worth noting that an overlarge VBO of 2D/3D perovskite can cause much higher bulk recombination of the perovskite layer, leading to the performance of devices with an overlarge VBO is almost independent of the *D*_it_ of the 2D/3D perovskite interface, as shown in Figure 2c. For an ultralow VBO case, although the energy cliff formed at the 2D/3D perovskite interface is beneficial for hole transport and electron obstruction, the adverse energy band bending near the 3D perovskite surface is not conducive to electron transport, resulting in the accumulation of electrons at the 2D/3D perovskite interface (Figure S4, Supporting Information). This makes it clear that the device with ultralow VBO is highly dependent on *D*_it_ of the 2D/3D perovskite interface compared with suitable and overlarge VBO cases, as demonstrated in Figure 2c,f,g, and S5, Supporting Information. Due to the unfavorable energy structure, an ultralow VBO at the 2D/3D perovskite interface will result in a reduced built‐in potential, which is the primary reason for the low *V*_oc_ as demonstrated by the *J–V* characteristics in Figure 2d.

In addition to the VBO, the influence of the bandgap and mobility of 2D perovskite on device performance is also investigated. The corresponding results are demonstrated in Figure 2h,i, Supporting Information, respectively. From Figure 2h, we can see that devices with an ultralow VBO are almost insensitive to the bandgap of 2D perovskite (*E*_g_2D_), while the overlarge and suitable VBO cases require a large *E*_g_2D_ (with a threshold bandgap value of 1.65 and 1.8 eV for the overlarge and suitable VBO cases, respectively) to achieve high device performance. As the 2D perovskite prepared in the experiment generally has a large bandgap (>1.8 eV), *E*_g_2D_ has a relatively minor effect on the device PCE, even if the energy band structure between 2D and 3D perovskite is overly mismatched. The dependence of the mobility of 2D perovskite on device performance is displayed in Figure 2i, which shows a similar trend with that of bandgap in Figure 2h, i.e., the device performance with an ultralow VBO (overlarge and suitable VBO) is almost independent (dependent) of the mobility of 2D perovskite. Moreover, we also investigate the device performance under different 2D perovskite thicknesses, as shown in Figure S6, Supporting Information. The results reveal that the suitable VBO is insensitive to 2D perovskite thickness. Overall, the VBO of 2D and 3D perovskite plays a crucial role in determining device performance, and the suitable VBO exhibits great tolerance for the bandgap, mobility, and thickness of 2D perovskite.

**Figure 3:**
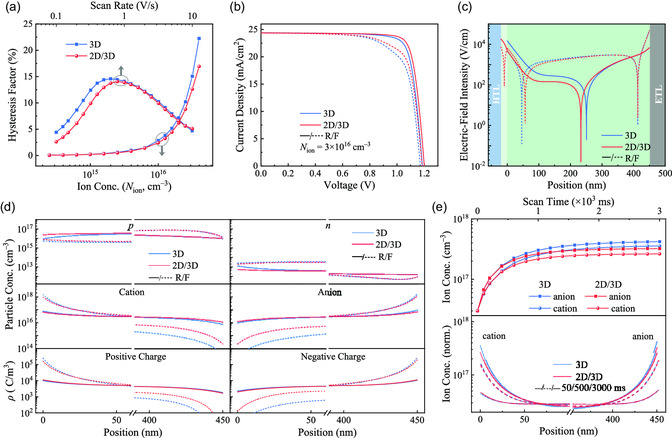
a) HFs of 3D and 2D/3D PSCs under the different ion concentrations and scan rates and b) the corresponding *J–V* curves of 3D and 2D/3D PSCs under *N*_ion_ of 3 × 10^16^ cm^−3^ and scan rate of 0.4 V s^−1^. Distributions of the position‐dependent c) electric field intensity and d) particle concentration and space charge density of 3D and 2D/3D PSCs at the reverse and forward scans under MPP‐like condition. e) The ion concentrations accumulated at the perovskite‐related interfaces as a function of scan time under a bias of 1 V, and the corresponding ion concentration profiles at the scan time of 50, 500, and 3000 ms.

In addition to enhancing device efficiency, the 2D/3D design has also gained widespread validity for its ability to improve device stability. Previous studies have attributed this phenomenon to the damage‐free deposition of subsequent films and the prevention of water and oxygen penetration.^[^ ^18^ ^]^ Ion migration, a widely accepted causal factor for hysteretic behavior and poor device stability, should be discussed in the context of 2D/3D PSCs.^[^ ^19^ ^]^ This section will elaborate on the effect of ion migration on the stability of 2D/3D PSCs under different ion concentrations and scan rates. As 2D perovskite is relatively stable, ion migration within 2D perovskite is thus not considered in this work, as shown in Figure S7, Supporting Information.^[^ ^20^ ^]^ To quantitatively evaluate the hysteresis effect, a typical indicator of the “Hysteresis Factor” (HF) is used, which can be defined as:

(1) $$\text{HF}=\frac{\text{PCE}(R)-\text{PCE}(F)}{\text{PCE}(R)}$$

where PCE(R)/PCE(F) represents the PCE value under reverse/forward voltage scan. The simulated HF values of 3D and 2D/3D PSCs under different scan rates and ion concentrations (*N*_ion_) are presented in **Figure** **3**a. It can be seen from these plots that if *N*_ion_ is less than 10^15^ cm^−3^, the hysteresis behavior of the two related cases with very low HF values is inapparent, while if *N*_ion_ is larger than 10^16^ cm^−3^ or the voltage scan rate is less than 1 V s^−1^, 2D/3D PSCs exhibit reduced hysteresis behavior with the appearance of a lower HF value compared to 3D PSCs. The 2D/3D PSC shows lower HF values than the 3D PSC, indicating that the 2D/3D design can suppress hysteresis behavior to a certain degree. The origin of the hysteresis behavior can be revealed by the *J–V* characteristics, as shown in Figure 3b, where the *N*_ion_ and scan rate were fixed at 3 × 10^16^ cm^−3^ and 0.4 V s^−1^, respectively. After careful observation, we can see that there is a significant difference in the *J–V* curves between forward and reverse scans for both cases, particularly relevant for the 3D PSC, implying that introducing a 2D/3D structure could partially suppress the hysteresis behavior of PSCs with better *J–V* characteristics.

As shown in Figure 1d, the 2D/3D PSC demonstrates a lower electric field within the perovskite layer compared to the 3D PSC due to the unique energy band structure of the 2D/3D configuration. It is well‐known that carriers (i.e., electrons and holes) and ions (i.e., cations and anions) driven by the built‐in electric field will move within the perovskite layer. Decreasing electric field intensity thus will concurrently diminish the driving force for the transport of both carriers and ions, thereby hindering the transport of carriers and ions to a certain extent. It is worth noting that the mobility of carriers typically falls within the range of 1–100 cm^2^ V^−1^ s, which is significantly higher than that of ions with the mobility of 10^−10^ to 10^−8^ cm^2^ V^−1^ s. This implies that the reduction in electric field in perovskite layer will have a more pronounced impact on the transport of ions than carriers. To validate this hypothesis, we perform a detailed simulation to study the migration/transport and accumulation behavior of carriers and mobile ions. Consistent with the results of the steady‐state electric field profiles in Figure 1d, the electric field distributions in Figure 3c under reverse and forward scans also suggest that the 3D PSC shows a slightly higher electric field than the 2D/3D PSC. This difference in electric field distribution results in a reduction in the accumulation of cations/anions at the perovskite‐related interfaces in 2D/3D PSCs compared to 3D PSCs, as confirmed by the ion and space charge density distributions in Figure 3d. It is clear from Figure 3d that the PSC under the forward scan exhibits more severe ion accumulation than the reverse scan, which is the primary cause for the more pronounced hysteresis behavior observed in Figure 3b. To further reveal the dynamic process of ion migration and accumulation, the time‐dependent ion concentrations at the perovskite‐related interfaces and the corresponding ion concentration profiles at typical scan times are investigated, as shown in Figure 3e. Irrespective of ion concentration, the 2D/3D PSCs exhibit rapid ion migration and accumulation processes. Moreover, the hysteresis effect at different VBO cases is also checked, as demonstrated in Figure S8, Supporting Information, which indicates that the energy band structure also influences the ion migration and accumulation process.

**Figure 4:**
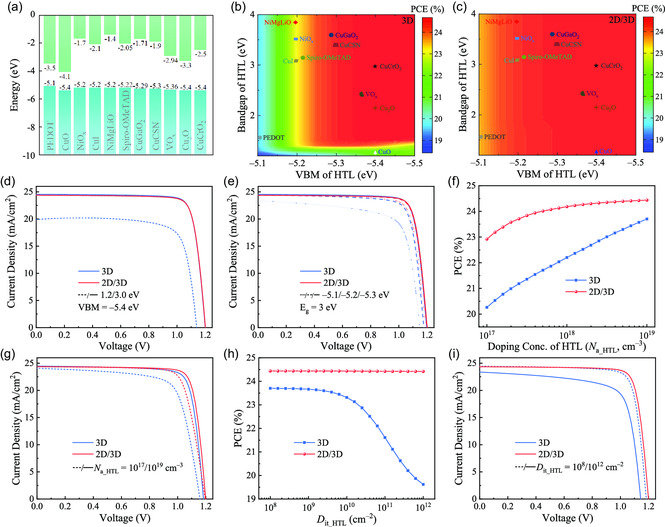
a) CBM and VBM of the commonly used HTLs extracted from the experiments. PCE contours of b) the 3D and c) 2D/3D PSCs as functions of VBM and bandgap of HTL. The corresponding *J–V* curves of 3D and 2D/3D PSCs under d) the bandgap of 1.2 and 3.0 eV (VBM = –5.4 eV) and e) the VBM of –5.1 and –5.3 eV (*E*_g_HTL_ = 3 eV). f) Dependence of the doping concentration of HTL on PCE of 3D and 2D/3D PSCs and g) the corresponding *J–V* curves under *N*_a_HTL_ of 10^17^ and 10^19^ cm^−3^. h) The variation of PCE of 3D and 2D/3D PSCs with *D*_it_HTL_ and i) the corresponding *J–V* curves under *D*_it_HTL_ of 10^8^ and 10^12^ cm^−2^, in which *N*_a_HTL_ is fixed at 10^19^ cm^−3^.

The aforementioned research mainly focuses on investigating the relationship between the 2D/3D structure and 2D material characteristics on the performance of 2D/3D PSCs. In this section, we will explore the dependence of the electrical parameters of HTL on device performance. The energy level alignment of the commonly used HTLs is illustrated in **Figure** **4**a, wherein the key parameters including conduction band minimum (CBM) and valence band maximum (VBM) are marked in the figure.^[^ ^21^, ^22^ ^]^ The PCE contours of 3D and 2D/3D PSCs under various VBM and bandgap of HTLs (*E*_g_HTL_) are depicted in Figure 4b,c, respectively. To facilitate comparison, the ratio of PCE values between 2D/3D and 3D PSCs is plotted in Figure S9, Supporting Information. After thorough examination, we conclude that 2D/3D PSCs can achieve higher PCE values than 3D PSCs, even with smaller *E*_g_HTL_ (i.e., <1.5 eV) or lower HTL VBM (i.e., <–5.1 eV), indicating the high tolerance of 2D/3D PSCs to the bandgap and VBM of HTLs. The typical HTLs are marked in Figure 4b,c, and the corresponding PCE values are shown in Figure S10, Supporting Information, which suggests that the 2D/3D design is effective for most HTLs to achieve higher device PCE. Moreover, the representative *J–V* curves under various bandgap and VBM of HTLs are plotted in Figure 4d,e, respectively. In contrast to 3D PSCs where the *J–V* curves are visibly deformed under a small *E*_g_HTL_ of 1.2 eV or low HTL VBM of –5.1 eV, 2D/3D PSCs exhibit negligible PCE degradation and can well‐maintain the shape of *J–V* curves. Moreover, the dependence of the doping concentration of HTL (*N*_a_HTL_) on the performance of 2D/3D PSCs is also studied, as demonstrated in Figure 4f,g. With the increase of *N*_a_HTL_ from 10^17^ to 10^19^ cm^−3^, the PCE of both 3D and 2D/3D PSCs gradually increases, but 2D/3D PSCs are less dependent on *N*_a_HTL_ compared to 3D PSCs. The investigation of the defect density of the HTL/perovskite interface (*D*_it_HTL_) on device performance is exhibited in Figure 4h,i. Apparently, the PCE of 3D PSCs remains relatively constant even under high *D*_it_HTL_ (e.g., 10^12^ cm^−2^), while a notable decrease in PCE for 3D PSCs can be seen especially when *D*_it_HTL_ > 10^10^ cm^−2^, revealing that the presence of the 2D/3D structure can almost shield the impact of HTL/perovskite interface defects on device performance. These observations can be attributed to the ability of 2D perovskite to strengthen the electric field intensity at the HTL/perovskite interface and suppress minority carrier concentration (i.e., electron), as shown in Figure 1d,g, respectively. Based on the preceding discussion, we can easily draw the conclusion that benefiting from the reduced minority carrier concentration in conjunction with the strengthened electric field at the HTL/perovskite interface, the 2D/3D PSCs possess a significant tolerance to the bandgap, VBM, doping concentration of HTL and HTL/perovskite interface defects, thereby relaxing the restrictions on HTL selectivity.

**Figure 5:**
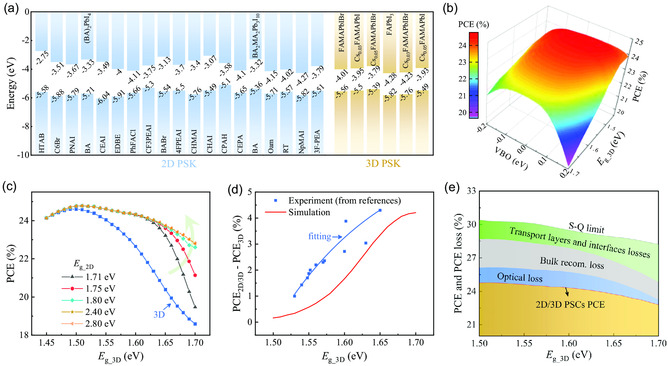
a) Conduction and valence band positions of the representative 2D and 3D perovskite extracted from refs. [15, 25, 26, 27, 28, 29, 30, 31, 32, 33, 34, 35, 36, 37]. b) PCE contour of 2D/3D PSCs under the various VBO of 2D/3D perovskite and *E*_g_3D_. c) The dependence of *E*_g_3D_ on PCE of 2D/3D PSCs under the different *E*_g_2D_ (the VBO is fixed at 0). d) The PCE difference between 2D/3D and 3D PSCs under the various *E*_g_3D_ (red line), in which the blue points fitted by blue line are the experiment results extracted from refs. [26, 27, 32, 34, 37, 38, 39, 40, 41, 42, 43, 44]. e) Repartition of PCE of 2D/3D PSCs and the corresponding PCE losses including HTL/ETL and interface recombination loss (caused by the energy mismatch and defects at the interfaces of 2D/3D perovskite, and the bulk of ETL/perovskite and HTL/perovskite), bulk recombination loss (caused by the perovskite defects), and optical loss (caused by reflection and parasitic absorption).

In the last section, the bandgap of 3D perovskite (*E*_g_3D_) will be discussed to further verify the wide applicability of the 2D/3D design for PSCs. Various 2D and 3D perovskite materials have been used to construct 2D/3D PSCs, and some of them are demonstrated in **Figure** **5**a, in which the conduction and valence band positions of these materials are labeled. To clarify the relationship between *E*_g_3D_ and the performance of 2D/3D PSCs, the PCE contour of 2D/3D PSCs under various VBOs of 2D/3D perovskite and *E*_g_3D_ is demonstrated in Figure 5b. For ease of comparison, the PCE contour of 2D/3D PSCs normalized by 3D PSCs is presented in Figure S11, Supporting Information. Although the PCE values of 2D/3D PSCs under different *E*_g_3D_ have the same trend as the VBO of 2D/3D perovskite changes (i.e., with the VBO of 2D/3D perovskite increasing from –0.2 to 0.2 eV, PCE values of 2D/3D PSCs under the same *E*_g_3D_ tend to increase first and then decrease), the wide‐bandgap PSCs yield lower PCE especially when the VBO of 2D/3D perovskite is mismatched, suggesting that wide‐bandgap PSCs put forward higher requirements for the VBO of 2D/3D perovskite.

Moreover, the dependence of *E*_g_3D_ on the performance of 2D/3D PSCs under different 2D perovskite bandgap (*E*_g_2D_) is demonstrated in Figure 5c, where the 3D PSC is also included as a reference. We can see from Figure 5c that the PCE values of 3D and 2D/3D PSCs initially increase with *E*_g_3D_ rising from 1.45 to 1.5 eV, and then decrease as *E*_g_3D_ continues to increase from 1.5 to 1.7 eV, yielding the best PCE at the 3D perovskite bandgap of 1.5 eV. This conclusion is consistent with the most experimental results. Interestingly, the 2D/3D PSCs have no noteworthy superiority in PCE compared to the 3D PSCs if *E*_g_3D_ is less than 1.5 eV. We speculate that the narrow‐bandgap PSCs have very low requirements for the energy level position of HTL, which can provide a strong electric field to reduce the minority carrier concentration at the interface, as shown in Figure S12, Supporting Information. Therefore, the 2D/3D structure is unnecessary. Consequently, the 3D and 2D/3D PSCs under the 3D perovskite bandgap of 1.5 eV have almost the same *J–V* characteristics, as demonstrated in Figure S13, Supporting Information. As the 3D perovskite bandgap increases from 1.5 to 1.7 eV, the PCE difference between 3D and 2D/3D PSCs increases especially for cases with a large 2D perovskite bandgap. This conclusion is supported by the experimental data shown in Figure 5d. As the wide‐bandgap PSCs are prone to cause energy band mismatch and severe interface recombination, as shown in Figure S12, Supporting Information, introducing 2D perovskite could ameliorate these drawbacks, resulting in a better *J–V* response (as see in Figure S13, Supporting Information) and improved device performance (as see in Figure S14, Supporting Information). In addition, the results in Figure 5c imply that the high‐efficiency wide‐bandgap PSCs with a 2D/3D perovskite structure also require a large 2D perovskite bandgap. This conclusion can be explained by the energy band structures and carrier distributions shown in Figure S15, Supporting Information, which suggest that the wide‐bandgap 2D perovskite with a low electron concentration could form a high energy barrier for suppressing electron transport and recombination. In a word, the 2D/3D perovskite structure has a more prominent effect on the promotion of the performance of wide‐bandgap PSCs, which is particularly relevant for cases with a large bandgap of 2D perovskite. It should be noticed that 3D PSCs with diverse bandgaps necessitate distinct HTLs for the fabrication of efficient devices, but this conclusion may extend to various other HTLs. Finally, to further analyze the origin of PCE deficits of the 2D/3D PSCs, the repartition of PCE losses under different *E*_g_3D_ is demonstrated in Figure 5e, which suggests that the efficiency of 2D/3D PSCs is still restricted by the imperfect optical management and the irrepressible nonradiative recombination at bulks and interfaces.

## 3 Conclusion

In summary, we clarified the working mechanisms and design principles of 2D/3D PSCs using a rigorous photoelectric simulation. Taking a *n‐i‐p* PSC as an example, we confirmed that inserting a 2D perovskite between the HTL and 3D perovskite could reduce the minority carrier concentration and improve the electric field intensity at the HTL/2D perovskite interface, thus significantly reducing the recombination at the HTL/perovskite interface and promoting the device performance. Owing to these advantages, the 2D/3D PSCs exhibit high tolerance to HTL/perovskite interface defects, and bandgap, VBM, doping concentration of HTL compared to the 3D counterparts. The simulation results also point out that the VBO of the 2D and 3D perovskite has a crucial impact on the performance of 2D/3D PSCs, and the performance of devices with a suitable VBO (i.e., ranging from –0.05 to 0.1 eV) can be well‐maintained even with a large range of bandgap, mobility, and thickness of the 2D perovskite. In addition, the 2D/3D PSCs with a relatively low electric field intensity at the 2D/3D interface tend to slow down ion migration and accumulation processes, leading to the suppression of hysteresis behavior especially in cases with high ion concentration or low voltage scan. This work is the first to account for the phenomenon that the 2D/3D perovskite design can inhibit ion migration of PSCs from the perspective of device physics. Furthermore, the impact of the 2D/3D perovskite design on the performance of devices with various 3D perovskite bandgaps was also studied, which reveals that the 2D/3D perovskite structure has a more prominent effect on wide‐bandgap PSCs. Overall, the simulation results and conclusions shed new light on the mixed‐dimensional PSCs, and provide valuable guidance for designing high‐efficiency PSCs.

## 4 Experimental Section

In this study, the photoelectric simulation was performed by finite element method based on the platform of COMSOL Multiphysics. First of all, the accurate spatial‐dependent distributions of electromagnetic field and the optical properties of the devices including the absorption, transmission, and reflection were obtained by solving Maxwell's equations under AM 1.5 G illumination:

(2) $$\nabla \times (\nabla \times E)=k_{0}\varepsilon_{c}E$$

(3) G(x,y,z)=∫g(x,y,z,λ)dλ

where *E* is the electromagnetic field intensity, *k*_0_ is the wave vector in free space, *ε*_c_ is the complex permittivity related to frequency, *G*(*x*, *y*, *z*) represents the spatial‐dependent distributions of photo‐generated carriers, and *g*(*x*, *y*, *z*, *λ*) represents the generation rate of carriers in position (*x*, *y*, *z*) for a specific wavelength *λ*.

Based on the photo‐generated carrier distributions in Figure S16, Supporting Information, the electrical simulation was carried out to address the behaviors of carrier transport and recombination by coupling carrier continuity equations, drift‐diffusion equations, and Poisson's equation:

(4) $$\frac{\partial n}{\partial t}=\frac{1}{q}\nabla \cdot J_{n}+G(x,y,z)-U;\;\;\frac{\partial p}{\partial t}=-\frac{1}{q}\nabla \cdot J_{p}+G(x,y,z)-U$$

(5) $$J_{n}=qn\mu_{n}(-\nabla \Phi )+qD_{n}\nabla n;\;\;J_{p}=qp\mu_{p}(-\nabla \Phi )-qD_{p}\nabla p$$

(6) $$\nabla^{2}\Phi =\frac{q}{\varepsilon }(n-p+N_{d}-N_{a})$$

where *n*/*p* is the electron/hole concentration, *q* is the electron charge, *J*_*n*_/*J*_p_ is the electron/hole current density, *μ*_*n*_/*μ*_p_ is the electron/hole mobility, *Φ* is the electrostatic potential, *D*_*n*_/*D*_p_ is the electron/hole diffusion coefficient, *N*_d_/*N*_a_ is the donor/acceptor doping concentration, and *U* is the total carrier recombination rate, which consists of three parts^[^ ^23^, ^24^ ^]^:

(7) $$U=U_{\text{rad}}+U_{\text{aug}}+U_{\text{SRH}}$$

(8)

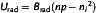



(9)

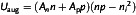



(10)

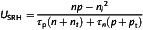

where *U*_rad_/*U*_aug_/*U*_SRH_ is the radiative/Auger/Shockley–Read–Hall (SRH) recombination rate, *n*_i_ is the intrinsic carrier concentration, *B*_rad_ is the radiative recombination rate, *A*_*n*_/*A*_p_ is the electron/hole Auger recombination rate, *τ*_*n*_/*τ*_p_ is the electron/hole recombination lifetime, and *n*_t_/*p*_t_ is the bulk electron/hole trap state concentration.

For ion migration, the ion current density (*J*_*i*_) can be expressed as:

(11) $$J_{i}=-D_{i}\nabla N_{i}-Z_{i}\mu_{i}FN_{i}\nabla \Phi$$

where *D*_*i*_ is ion diffusion coefficient, *N*_*i*_ is ion concentration, *Z*_*i*_ is ion charge number, *μ*_*i*_ is ion mobility, and *F* is the Faraday constant.

The simulation details were summarized as below: 1) a low electrical conductivity of 2D perovskite with the electron/hole mobility of 0.1/0.1 cm^2^ V^−1^ s^−1^ is considered compared with 3D perovskite with the electron/hole mobility of 12.5/7.5 cm^2^ V^−1^ s^−1^; 2) the variation of VBO between 2D and 3D perovskite is realized by regulating the electron affinity of 2D perovskite, where the bandgap of 2D perovskite is fixed, or the electron affinity is fixed when regulating the 2D perovskite bandgap, the results obtained by both methods are same; 3) an ideal Ohmic contact is considered at electrode/transport layer interfaces; 4) for SRH recombination, the trap states of all energies are approximated into a single energy at the middle level of the bandgap with a trap occupancy of 0.5; 5) as for the ion migration, an uniform ion distribution within 3D perovskite is supposed at the beginning of voltage scan; and 6) in terms of the various HTL and perovskite materials, the main difference between them is the energy band structure. Moreover, the simulation results are obtained based on the following assumptions: 1) the excitons immediately dissociate into free electron and hole pairs; 2) the hot carrier effect is not considered; 3) the device temperature remains constant (*T* = 300 K); and 4) photon recycling effect is considered with the modified radiation recombination coefficient.

## Conflict of Interest

The authors declare no conflict of interest.

## Author Contributions

Y.Z.: Methodology, software, investigation, data curation, and writing—original draft; Z.Y.: Methodology, conceptualization, supervision, funding acquisition, investigation, and writing— review and editing; T.M.: Software, data curation; Z.A.: Software, visualization; Y.B.: Data curation; L.S.: Visualization; L.Q.: Supervision; G.C.: Writing—review and editing; C.W.: Methodology, supervision, and funding acquisition; X.L.: Methodology, conceptualization, supervision, funding acquisition, investigation, and writing—review and editing.

## Supporting information

Supplementary Material

## Data Availability

The data that support the findings of this study are available from the corresponding author upon reasonable request.
