# Supplementary material for: Device Physics and Design Principles of Mixed‐Dimensional Heterojunction Perovskite Solar Cells
Source: Small Sci. 2024 Jan 20;4(3):2300188. doi: 10.1002/smsc.202300188 (PMC11935289; doi:10.1002/smsc.202300188)
Supplement: Supplementary file 1 — Supplementary Material [file SMSC-4-2300188-s001.pdf]

Supporting Information

Device Physics and Design Principles of Mixed-Dimensional Heterojunction Perovskite Solar Cells

Yuqi Zhang [a,b], Zhenhai Yang [a,b,*], Tianshu Ma [a,b], Zhenhai Ai [a,b], Yining Bao [a,b], Luolei Shi [a,b], Linling Qin [a,b], Guoyang Cao [a,b], Changlei Wang [a,b], and Xiaofeng Li [a,b,*]

[a]School of Optoelectronic Science and Engineering & Collaborative Innovation Center of Suzhou Nano Science and Technology, Soochow University, Suzhou 215006, China

[b]Key Lab of Advanced Optical Manufacturing Technologies of Jiangsu Province & Key Lab of Modern Optical Technologies of Education Ministry of China, Soochow University, Suzhou 215006, China

[*]Corresponding Authors:

Z. Y. (yangzhenhai@nimte.ac.cn); X. L. (xfli@suda.edu.cn)

Table S1. A summary of the representative 2D/3D PSCs.

Year	Institution	Type	Spacer Cation Compound	Structure	PCE	Ref.
2019.03	KRICT	n-i-p	HTAB	FTO/d-TiO_2/mp-TiO_2/$(FAPbI_3)_{0.95}(MAPbBr_3)_{0.05}$/HTAB/Spiro-OMeTAD/Au	23.30%	[1]
2019.04	CAS	n-i-p	PEAI	ITO/SnO_2/$FA_{0.92}MA_{0.08}PbI_3$/PEAI/Spiro-OMeTAD/Au	23.32%	[2]
2019.06	MIT	n-i-p	C_6Br	FTO/c-TiO_2/m-TiO_2/$(FAPbI_3)_{0.92}(MAPbBr_3)_{0.08}$/$C_6Br$/Spiro-OMeTAD/Au	23.40%	[3]
2020.10	KAUST	p-i-n	Oam	ITO/PTAA/$Cs_{0.05}(FA_{0.92}MA_{0.08})_{0.95}Pb(I_{0.92}Br_{0.08})_3$/Oam/$C_{60}$/Cu	23%	[4]
2020.11	ANU	n-i-p	DIAC	FTO/m-TiO_2/PMMA/$Cs_{0.07}Rb_{0.03}FA_{0.765}MA_{0.135}PbI_{2.55}Br_{0.45}$/DIAC/Spiro-OMeTAD/Au	23.27%	[5]
2021.01	KU	n-i-p	BA	FTO/SnO_2/$(FAPbI_3)_{0.95}(MAPbBr_3)_{0.05}$/BA/Spiro-OMeTAD/Au	24.59%	[6]
2021.01	CAS	n-i-p	oFPEAI	FTO/SnO_2/$FA_{0.92}MA_{0.08}Pb(I_{0.92}Br_{0.08})_3$/oFPEA/Spiro-OMeTAD/Ag	23.80%	[7]
2021.01	ISM-CNR	n-i-p	CEAI	ITO/TiO_2/$Cs_{0.05}MA_{0.1}FA_{0.85}PbI_{2.9}Br_{0.1}\cdot 0.05PbI_2$/CEAI/Spiro-OMeTAD/Au	23.57%	[8]
2021.04	RECAST	n-i-p	PhFACl	ITO/SnO_2/FAMAPbICl/PhFACl/Spiro-OMeTAD/Au	23.36%	[9]
2021.05	SNNU	n-i-p	CF_3PEAI	FTO/TiO_2/$FA_{0.85}MA_{0.15}PbI_3$/CF_3PEAI/Spiro-OMeTAD/Au	23.10%	[10]
2021.07	RISE	n-i-p	BABr	ITO/SnO_2/$Cs_{0.03}(FA_{0.97}MA_{0.03})_{0.97}Pb(I_{0.97}Br_{0.03})_3$/BABr/Spiro-OMeTAD/Au	23.78%	[11]
2021.09	POSTEC	n-i-p	CHMAI	FTO/SnO_2/$FAPbI_3$/CHMAI/Spiro-	23.91%	[12]

	H			OMeTAD/Au		
2021.09	POSTECH	n-i-p	CHAI	FTO/SnO_2/$FAPbI_4$/CHAI/Spiro-OMeTAD/Au	23.10%	[12]
2021.11	CAS	n-i-p	MT-Im	ITO/SnO_2/$(Cs_{0.03}FA_{0.97}PbI_3)_{0.95}(MAPbBr_3)_{0.05}$/MT-Im/Spiro-OMeTAD/Au	24.07%	[13]
2021.11	EPFL	n-i-p	$PDEAI_2$	FTO/TiO_2/SnO_2/CsFAMAPbIBrCl/$PDEAI_2$/Spiro-OMeTAD/Au	23.90%	[14]
2021.12	CDLCEM	n-i-p	neoPACl	ITO/SnO_2/$FA_{1-x}MA_xPbI_3$/neoPACl/Spiro-OMeTAD/Au	23.35%	[15]
2022.01	NREL	n-i-p	$BDAI_2$	FTO/SnO_2/$FA_{0.97}MA_{0.03}PbI_{2.91}Br_{0.09}$/$BDAI_2$/Spiro-OMeTAD/Au	24.70%	[16]
2022.02	KAUST	p-i-n	RT	ITO/2PACz/$Cs_{0.03}(FA_{0.90}MA_{0.10})_{0.97}PbI_3$/RT/$C_{60}$/Ag	24.30%	[17]
2022.03	RECAST	p-i-n	NpMAI	ITO/SnO_2/$FA_{0.85}MA_{0.15}PbI_3$/NpMAI/Spiro-OMeTAD/MoO_x/Ag	24.37%	[18]
2022.03	KAIST	n-i-p	CEPA	FTO/SnO_2/FAMAPbBrI/CEPA/PTAA/Au	23.60%	[19]
2022.03	UofT	p-i-n	3F-PEA	ITO/NiO_x/$Cs_{0.05}FA_{0.85}MA_{0.1}PbI_3$/3F-PEA/$C_{60}$/Ag	23.30%	[20]
2022.09	Rice	n-i-p	BA	ITO/SnO_2/$Cs_{0.05}(MA_{0.10}FA_{0.85})Pb(I_{0.90}Br_{0.10})_3$/BA/Spiro-OMeTAD/Au	24.50%	[21]

Table S2. The key parameters used for this simulation. [21-23]

Parameters	SnO_2	3D PSCs	2D/3D PSCs		Spiro-OMeTAD
		PSK	3D PSK	2D PSK	
Relative permittivity	9	25	25	25	3
Electron affinity (eV)	4	3.79	3.79	3.32	2.05
Bandgap (eV)	3.6	1.58	1.58	2.04	3.17
Thickness (nm)	50	450	430	20	150
Doping conc. (cm^{-3})	10^{19}	3.5×10^{16}	3.5×10^{16}	10^{17}	5×10^{18}
Mobility of electron (cm^2/Vs)	0.01	12.5	12.5	0.1	0.0003
Mobility of hole (cm^2/Vs)	0.01	7.5	7.5	0.1	0.0003
SRH life time (μs)	1	/	/	/	1
Bulk defect conc. (cm^{-3})	/	10^{15}	10^{15}	10^{16}	/
Radiative recombination coefficient (cm^3/s^{-1})	0	3.27×10^{-11}	3.27×10^{-11}	3.27×10^{-11}	/

Table S3. The photovoltaic parameters of the related devices extracted from Figure 1b.

Parameters	J_{sc} (mA/cm^2)	V_{oc} (V)	*FF* (%)	PCE (%)
2D/3D PSC (Experiment)	24.34	1.20	84.0	24.5
2D/3D PSC (Simulation)	24.36	1.20	83.4	24.4
3D PSC (Simulation)	24.39	1.18	80.9	23.3

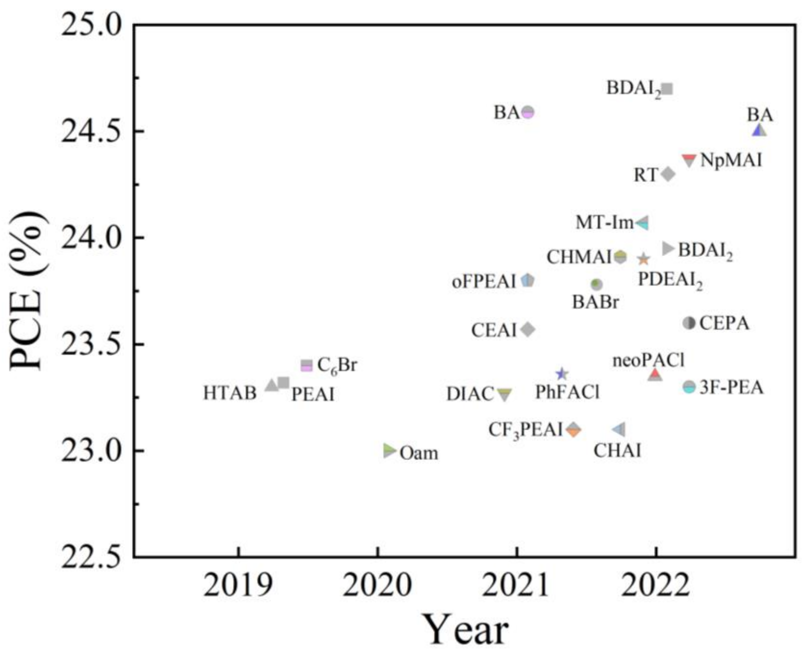

Figure S1. Statical graph of efficiency and the corresponding 2D materials of 2D/3D PSCs extracted from Table S1.

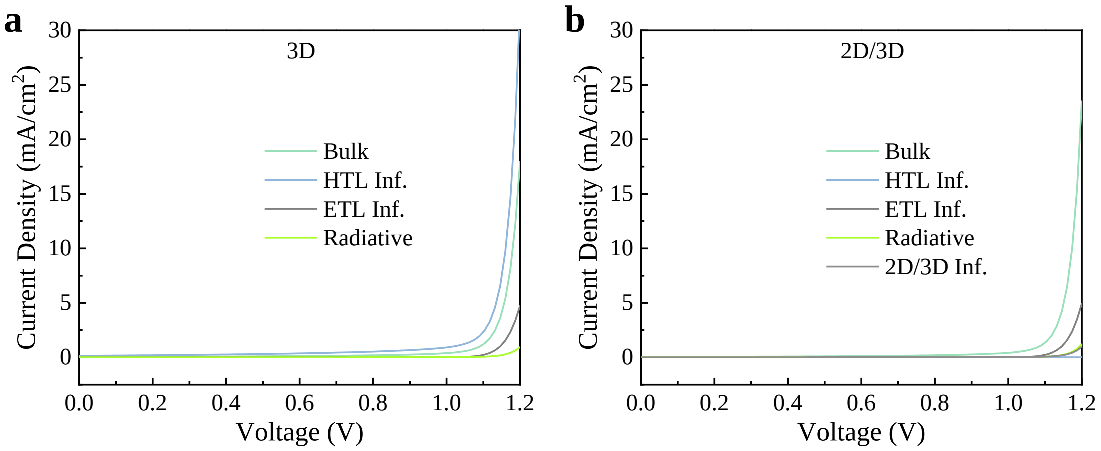

Figure S2. Current losses contributed from the different sources for (a) 3D and (b) 2D/3D PSCs, respectively.

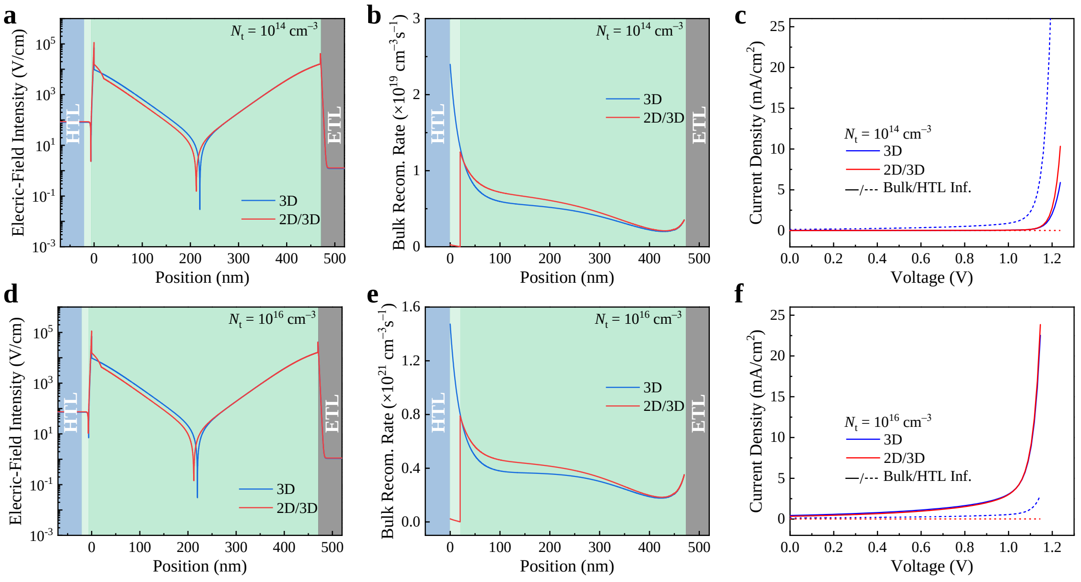

Figure S3. (a)/(d) Electric-field and (b)/(e) bulk recombination distributions, and (c)/(f) current density losses of bulk and HTL/perovskite interface for the 3D and 2D/3D PSCs under $N_t = 10^{14}/10^{16}$ cm^{-3}.

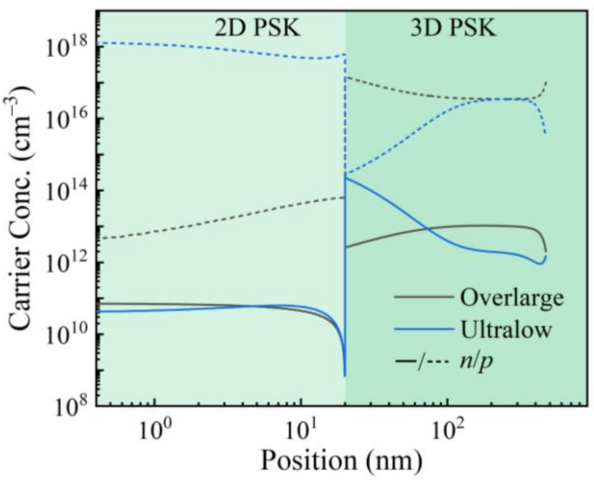

Figure S4. Carrier distributions of the 3D and 2D/3D PSCs under the overlarge and ultralow VBO conditions.

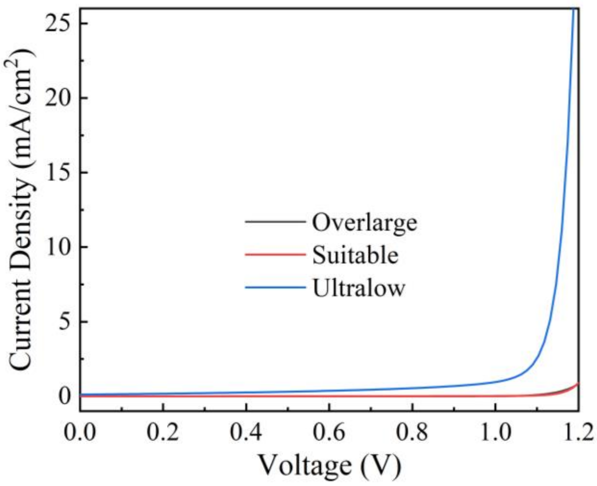

Figure S5. Current density losses at 2D/3D interface for 2D/3D PSCs under the different VBO conditions.

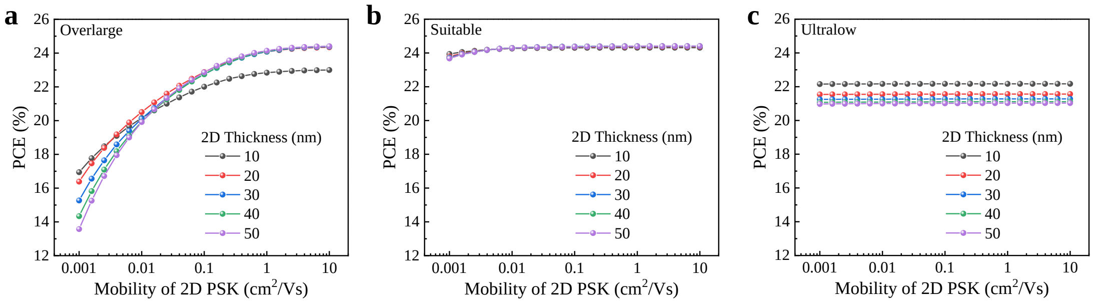

Figure S6. Dependence of mobility and thickness of 2D perovskite on PCE for the (a) overlarge, (b) suitable and (c) ultralow VBO cases.

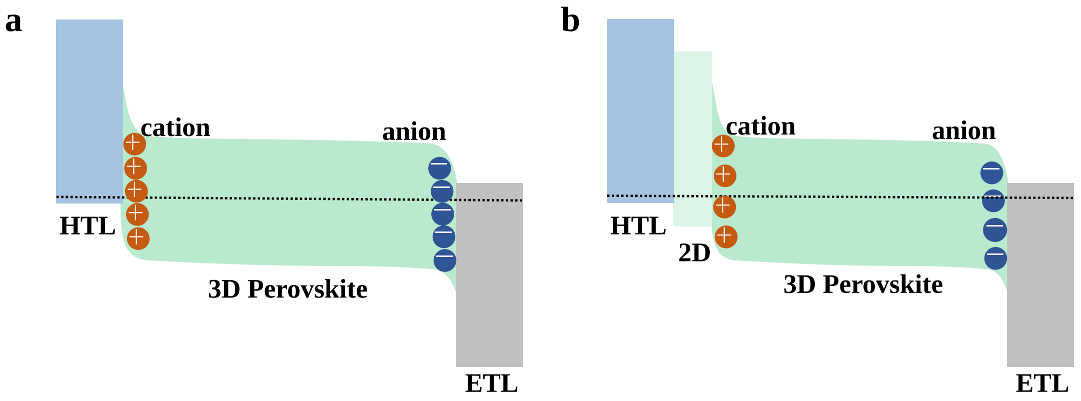

Figure S7. Energy diagrams of (a) 3D and (b) 2D/3D PSCs under equilibrium condition.

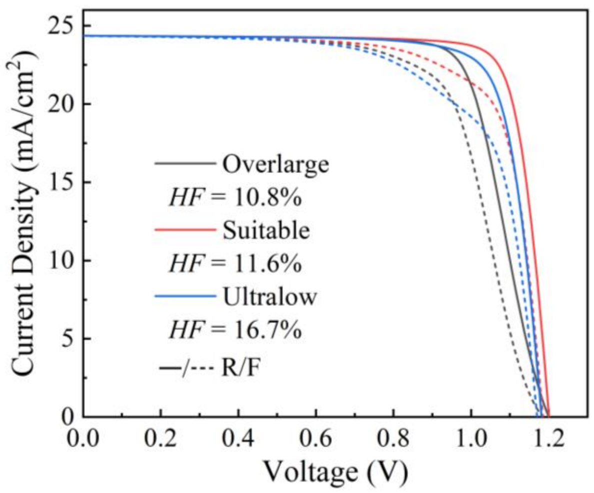

Figure S8. *J-V* curves with hysteresis effect under the different VBO conditions, where N_{ion} and scan rate are fixed at 3×10^{16} cm^{-3} and 0.4 V/s, respectively.

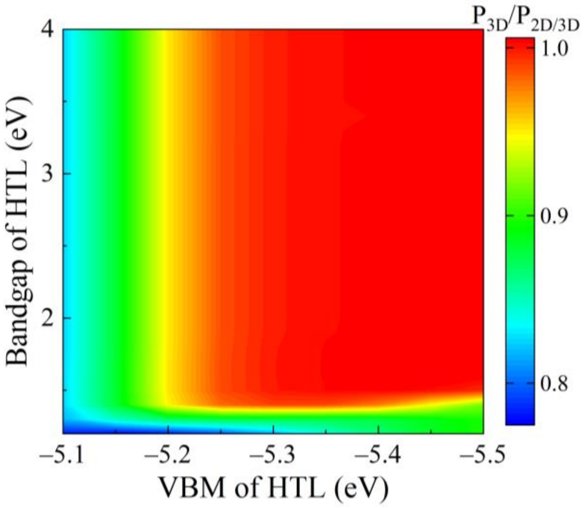

Figure S9. The ratio of PCE values between 2D/3D and 3D PSCs in Figure 4b and 4c.

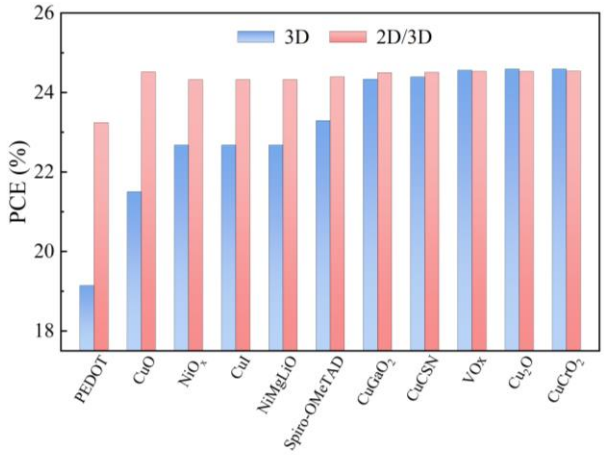

Figure S10. The histogram of PCE values of 3D and 2D/3D PSCs with the typical HTLs.

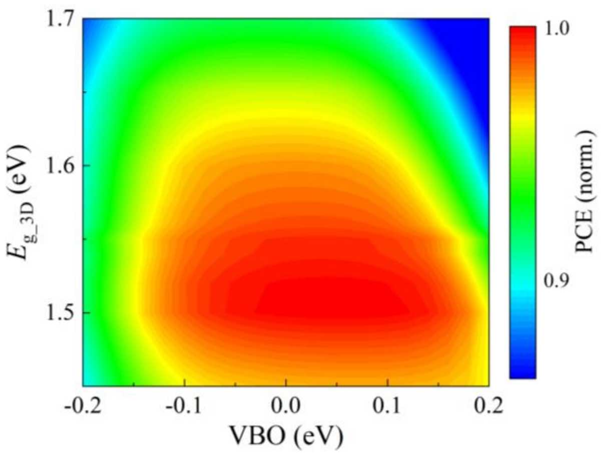

Figure S11. PCE contour of 2D/3D PSCs normalized by 3D PSCs under the various VBO of 2D/3D perovskite and 3D perovskite bandgap.

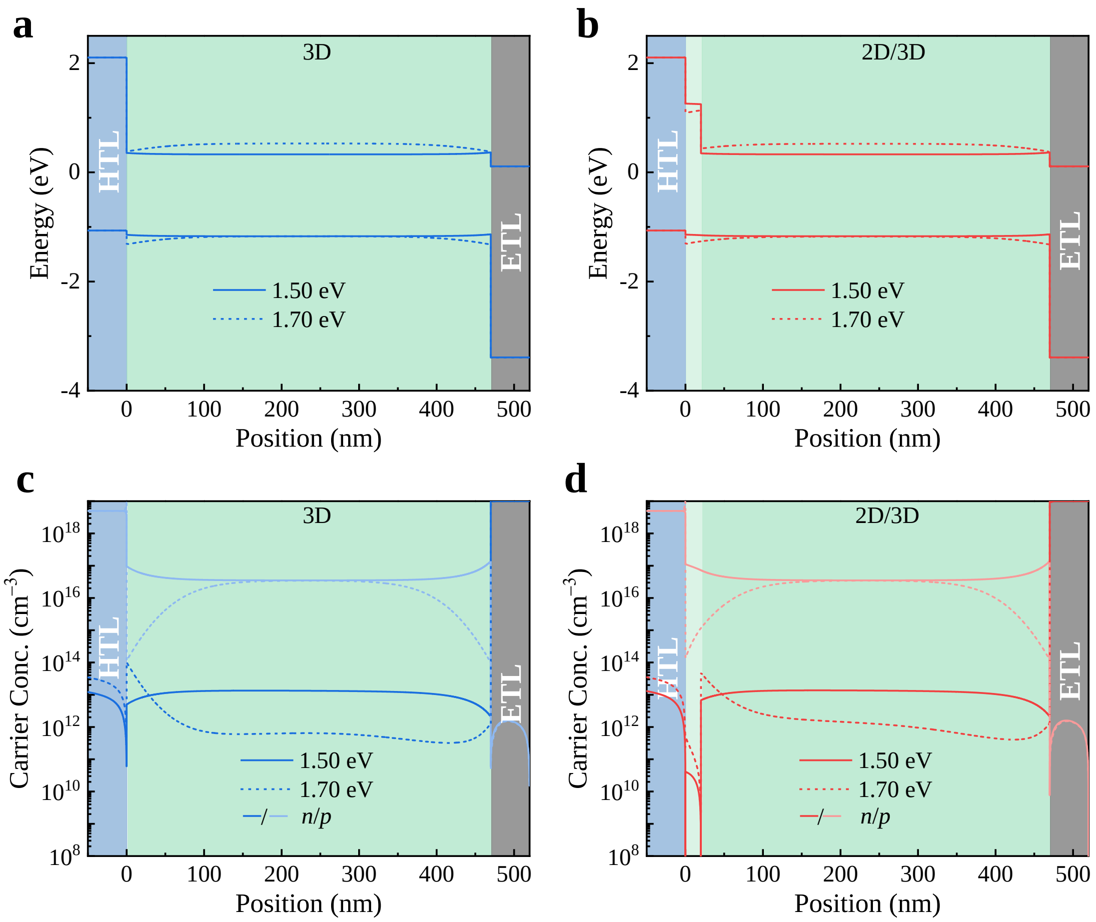

Figure S12. (a)-(b) Energy band structures, and (c)-(d) carrier concentrations under the 3D perovskite bandgap of 1.50 and 1.70 eV for 3D and 2D/3D PSCs.

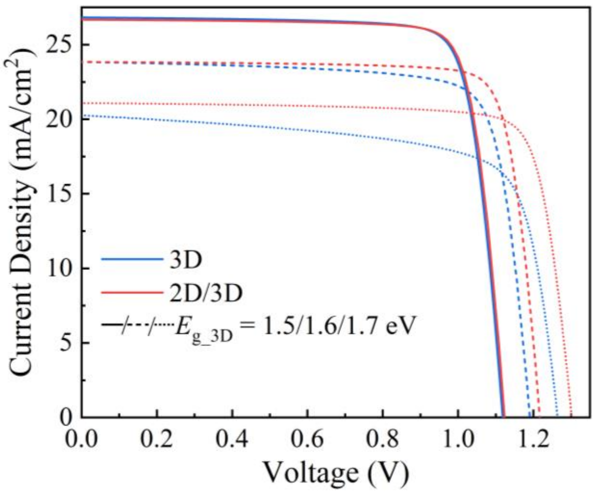

Figure S13. *J*-*V* curves of 3D and 2D/3D PSCs under the 3D perovskite bandgap of 1.5, 1.6 and 1.7 eV in Figure 5c (where the 2D perovskite bandgap is fixed at 2.40 eV).

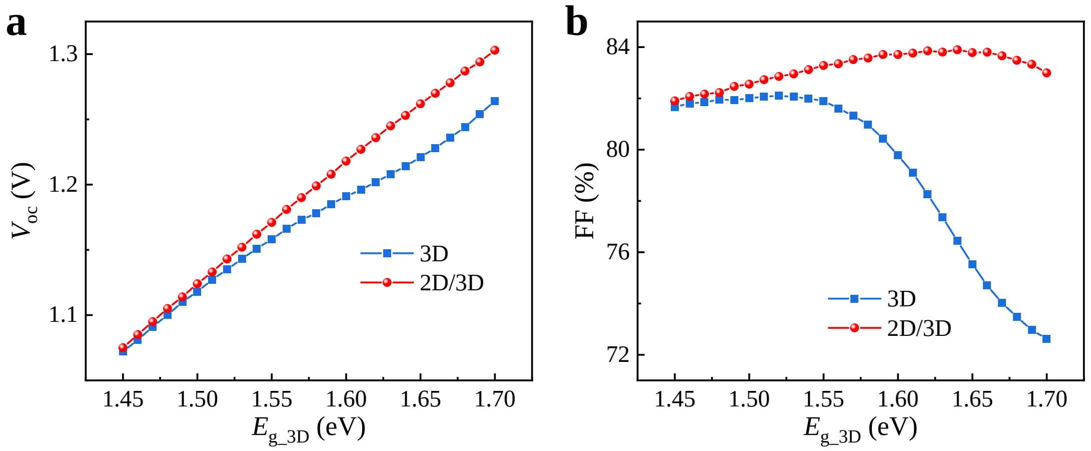

Figure S14. (a) V_{oc} and (b) *FF* of 3D and 2D/3D PSC as a function of E_{g_3D}.

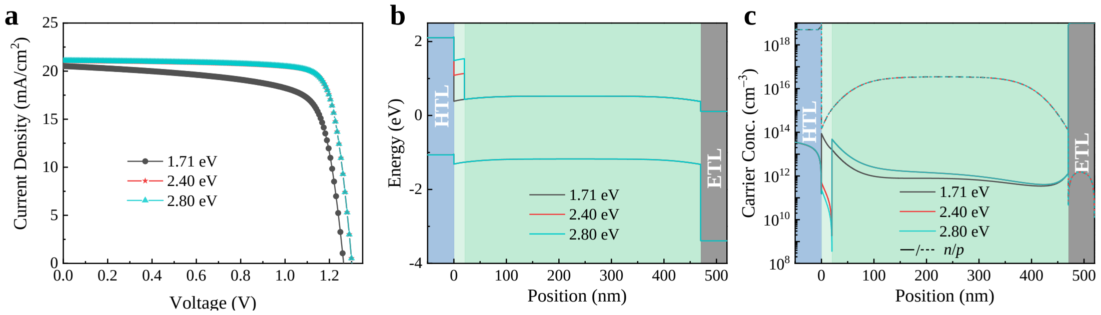

Figure S15. (a) *J-V* curves, (b) energy band structures, and (c) carrier concentrations of 2D/3D PSs under the 2D perovskite bandgap of 1.71, 2.40 and 2.80 eV, where the 3D perovskite bandgap is fixed at 1.70 eV.

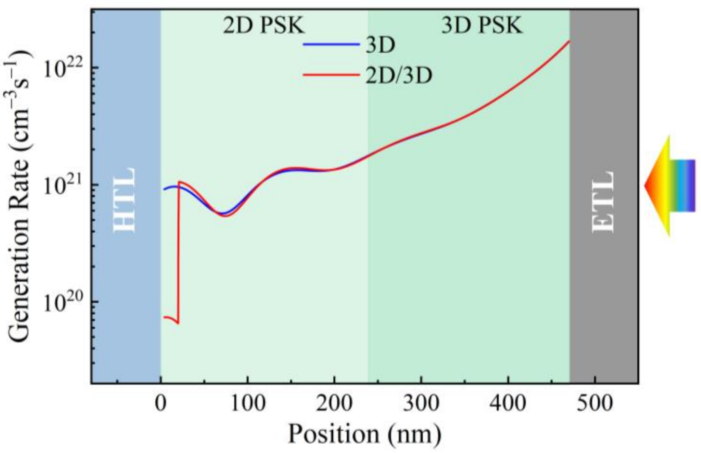

Figure S16. Carrier generation rates within perovskite for 3D and 2D/3D PSCs.

References

[1] E. H. Jung, N. J. Jeon, E. Y. Park, C. S. Moon, T. J. Shin, T. Y. Yang, J. H. Noh and J. Seo, *Nature* **2019**, *567*, 511.

[2] Q. Jiang, Y. Zhao, X. Zhang, X. Yang, Y. Chen, Z. Chu, Q. Ye, X. Li, Z. Yin and J. You, *Nat. Photonics* **2019**, *13*, 460.

[3] J. J. Yoo, S. Wieghold, M. C. Sponseller, M. R. Chua, S. N. Bertram, N. T. P. Hartono, J. S. Tresback, E. C. Hansen, J.-P. Correa-Baena, V. Bulović, T. Buonassisi, S. S. Shin and M. G. Bawendi, *Energy Environ. Sci.* **2019**, *12*, 2192.

[4] X. Zheng, Y. Hou, C. Bao, J. Yin, F. Yuan, Z. Huang, K. Song, J. Liu, J. Troughton, N. Gasparini, C. Zhou, Y. Lin, D.-J. Xue, B. Chen, A. K. Johnston, N. Wei, M. N. Hedhili, M. Wei, A. Y. Alsalloum, P. Maity, B. Turedi, C. Yang, D. Baran, T. D. Anthopoulos, Y. Han, Z.-H. Lu, O. F. Mohammed, F. Gao, E. H. Sargent and O. M. Bakr, *Nat. Energy* **2020**, *5*, 131.

[5] M. A. Mahmud, T. Duong, Y. Yin, J. Peng, Y. Wu, T. Lu, H. T. Pham, H. Shen, D. Walter, H. T. Nguyen, N. Mozaffari, G. D. Tabi, Y. Liu, G. Andersson, K. R. Catchpole, K. J. Weber and T. P. White, *Small* **2020**, *16*, 2005022.

[6] Y.-W. Jang, S. Lee, K. M. Yeom, K. Jeong, K. Choi, M. Choi and J. H. Noh, *Nat. Energy* **2021**, *6*, 63.

[7] Q. Zhou, Y. Gao, C. Cai, Z. Zhang, J. Xu, Z. Yuan and P. Gao, *Angew. Chem. Int. Ed.* **2021**, *60*, 8303.

[8] B. Yang, J. Suo, F. Di Giacomo, S. Olthof, D. Bogachuk, Y. Kim, X. Sun, L. Wagner, F. Fu, S. M. Zakeeruddin, A. Hinsch, M. Gratzel, A. Di Carlo and A. Hagfeldt, *ACS Energy Lett.* **2021**, *6*, 3916.

[9] T. Liu, J. Guo, D. Lu, Z. Xu, Q. Fu, N. Zheng, Z. Xie, X. Wan, X. Zhang, Y. Liu and Y. Chen, *ACS Nano* **2021**, *15*, 7811.

[10] Y. Cai, J. Wen, Z. Liu, F. Qian, C. Duan, K. He, W. Zhao, S. Zhan, S. Yang, J. Cui and S. Liu, *J. Energy Chem.* **2022**, *65*, 480.

[11] G. Yang, Z. Ren, K. Liu, M. Qin, W. Deng, H. Zhang, H. Wang, J. Liang, F. Ye, Q. Liang, H. Yin, Y. Chen, Y. Zhuang, S. Li, B. Gao, J. Wang, T. Shi, X. Wang, X. Lu, H. Wu, J. Hou, D. Lei, S. K. So, Y. Yang, G. Fang and G. Li, *Nat. Photonics* **2021**, *15*, 681.

[12] S. Jeong, S. Seo, H. Yang, H. Park, S. Shin, H. Ahn, D. Lee, J. H. Park, N. G. Park and H.

Shin, *Adv. Energy Mater.* **2021**, *11*, 2102236.

[13] G. Liu, H. Zheng, J. Ye, S. Xu, L. Zhang, H. Xu, Z. Liang, X. Chen and X. Pan, *ACS Energy Lett.* **2021**, *6*, 4395.

[14] C. Liu, Y. Yang, K. Rakstys, A. Mahata, M. Franckevicius, E. Mosconi, R. Skackauskaite, B. Ding, K. G. Brooks, O. J. Usiobo, J. N. Audinot, H. Kanda, S. Driukas, G. Kavaliauskaite, V. Gulbinas, M. Dessimoz, V. Getautis, F. De Angelis, Y. Ding, S. Dai, P. J. Dyson and M. K. Nazeeruddin, *Nat. Commun.* **2021**, *12*, 6394.

[15] X. Liu, T. Webb, L. Dai, K. Ji, J. A. Smith, R. C. Kilbride, M. Yavari, J. Bi, A. Ren, Y. Huang, Z. Wang, Y. Shen, G. Shao, S. J. Sweeney, S. Hinder, H. Li, D. G. Lidzey, S. D. Stranks, N. C. Greenham, S. R. P. Silva and W. Zhang, *Energy Environ. Mater.* **2022**, *5*, 670.

[16] F. Zhang, S. Y. Park, C. Yao, H. Lu, S. P. Dunfield, C. Xiao, S. Uličná, X. Zhao, L. Du Hill and X. Chen, *Science* **2022**, *375*, 71.

[17] R. Azmi, E. Ugur, A. Seitkhan, F. Aljamaan, A. S. Subbiah, J. Liu, G. T. Harrison, M. I. Nugraha, M. K. Eswaran, M. Babics, Y. Chen, F. Xu, T. G. Allen, A. U. Rehman, C. L. Wang, T. D. Anthopoulos, U. Schwingenschlogl, M. De Bastiani, E. Aydin and S. De Wolf, *Science* **2022**, *376*, 73.

[18] T. Zhou, Z. Xu, R. Wang, X. Dong, Q. Fu and Y. Liu, *Adv. Mater.* **2022**, *34*, 2200705.

[19] S. J. Sung, J. Im, G. Kim, C. S. Moon, J. J. Yoo, S. S. Shin, N. J. Jeon, B. S. Ma, D. J. Kim, T. S. Kim and J. Seo, *Adv. Energy Mater.* **2022**, *12*, 2200758.

[20] H. Chen, S. Teale, B. Chen, Y. Hou, L. Grater, T. Zhu, K. Bertens, S. M. Park, H. R. Atapattu, Y. Gao, M. Wei, A. K. Johnston, Q. Zhou, K. Xu, D. Yu, C. Han, T. Cui, E. H. Jung, C. Zhou, W. Zhou, A. H. Proppe, S. Hoogland, F. Laquai, T. Filleter, K. R. Graham, Z. Ning and E. H. Sargent, *Nat. Photonics* **2022**, *16*, 352.

[21] S. Sidhik, Y. Wang, M. De Siena, R. Asadpour, A. J. Torma, T. Terlier, K. Ho, W. Li, A. B. Puthirath, X. Shuai, A. Agrawal, B. Traore, M. Jones, R. Giridharagopal, P. M. Ajayan, J. Strzalka, D. S. Ginger, C. Katan, M. A. Alam, J. Even, M. G. Kanatzidis and A. D. Mohite, *Science* **2022**, *377*, 1425.

[22] Y. An, A. Shang, G. Cao, S. Wu, D. Ma and X. Li, *Sol. RRL* **2018**, *2*, 1870227.

[23] Y. An, C. Wang, G. Cao and X. Li, *ACS Nano* **2020**, *14*, 5017.
